# Current News

**Published:** 1902-02

**Authors:** 


### Current News.

### NEW JERSEY STATE DENTAL SOCIETY.

The Committee on Exhibits desires to announce that at the thirty-second annual meeting of the New Jersey State Dental Society, to be held as usual, in the "Auditorium," Asbury Park, July 16, 17, and 18, 1902, the large room, which is especially adapted for exhibition purposes, will be devoted exclusively to the exhibits. Every advantage is here offered for a great display, with all the conveniences necessary for such an exhibition.

This will undoubtedly be a "big year," and especially so from the exhibit stand-point, as many exhibitors have already written to secure the space generally selected by them.

A great inducement offered to all exhibitors is the fact that at last year's meeting over five hundred dentists registered at the entrance to the Exhibit Hall.

The names of the exhibitors selecting space prior to the programme going to press will be mentioned therein, together with the nature of their display.

It is earnestly requested that those desiring space communicate with the chairman at an early date.

Frank L. Hindle,
*Chairman of the Committee on Exhibits.*

New Brunswick, N. J.

### ODONTOGRAPHIC SOCIETY OF CHICAGO.

At the annual meeting of the Odontographic Society, held Monday, December 16, 1901, the following officers were elected for the ensuing year:

President, C. N. Johnson; Vice-President, W. T. Reeves; Secretary, Frank H. Zinn; Treasurer, Geo. N. West.

*Board of Directors.*—Geo. B. Perry, F. E. Roach, L. O. Green.

*Board of Censors.*— F. B. Noyes, W. Girling, D. A. Hare.

*Programme Committee.*—C. E. Bentley, L. S. Tenney, H. J. Goslee.

The Fifteenth Anniversary of the Society will occur in December, 1902. It is the intention of the Society to celebrate the event by giving a rousing Clinic, extending over two days, and a meeting that will be memorable in its history.

Prominent members from all parts of the country are to be invited to be present. The Programme Committee have already commenced plans for one of the most notable meetings that has ever been held in this country.

FRANK H. ZINN,
*Secretary.*

100 STATE STREET, CHICAGO.

---

### A BILL TO ADD DENTAL SURGEONS TO THE NAVY.

IN the Senate of the United States, January 8, 1902, Mr. Pettus introduced the following bill, which was read twice and referred to the Committee on Naval Affairs:

"A BILL TO ADD DENTAL SURGEONS TO THE MEDICAL CORPS OF THE NAVY.

"*Be it enacted by the Senate and House of Representatives of the United States of America in Congress assembled,* That to the Medical Corps of the Navy there shall be attached a corps of dental surgeons to serve the officers, enlisted men, and boys in the naval military service and training schools, which corps shall not exceed in number the proportion of one to one thousand authorized by law for said service and schools.

"The said dental corps shall consist of three grades, designated assistant dental surgeon, passed assistant dental surgeon, and dental surgeon, and with respect to rank, pay, and allowances to promotion within said dental corps the grades named shall correspond to the grades of the Medical Corps designated assistant surgeon, passed assistant surgeon, and surgeon, respectively.

"SEC. 2.—That original appointments shall be made to the grade of assistant dental surgeon and the appointees must be citizens of the United States, between twenty-three and thirty-three years of age, graduates of standard dental colleges, with not less than two years' subsequent experience in practice, of good moral character, of unquestionable professional repute, and shall be required to pass a satisfactory physical and professional examination: *Provided,* That there shall first be selected a member of the dental profession who is a graduate of a standard dental college and whose aptitude and experience evidence eminent fitness for conducting the professional examinations herein provided for, and for otherwise assisting in organizing, equipping, and supervising the operations of the corps, who shall be first appointed to the grade of dental surgeon."

---

### SOUTHERN BRANCH, NATIONAL DENTAL ASSOCIATION.

The fifth annual meeting of the Southern Branch of the National Dental Association will be held at Atlanta, February 18, 1902. The Association will be in session four days. Atlanta is now the best located and equipped city in the South for holding such a meeting. This fact assures a large attendance.

The Southeastern Passenger Association will give a rate of one and one-third fare for the round trip on the certificate plan. They will also give an extension of ten days after the close of the meeting so as to enable those attending the Association to visit the Charleston Exposition. A cheap round-trip ticket from Atlanta to Charleston will be on sale. An effort will be made to have a special train for the dentists. Delegates living beyond the territory of the Southeastern Passenger Association can purchase a winter tourist ticket to Charleston by way of Atlanta, as these tickets have a stop-off privilege of fifteen days at Atlanta.

H. H. Johnson, *President,*
Macon, Ga.
C. L. Alexander, *Corresponding Secretary,*
Charlotte, N. C.

